# Gait phase recognition of children with cerebral palsy via deep learning based on IMU data from a soft ankle exoskeleton

**DOI:** 10.3389/fbioe.2025.1679812

**Published:** 2025-09-10

**Authors:** Zhi Pang, Zewei Li, Ying Li, Bingshan Hu, Qiu Wang, Hongliu Yu, Wujing Cao

**Affiliations:** ^1^ School of Health Science and Engineering, University of Shanghai for Science and Technology, Shanghai, China; ^2^ Pingshan County People’s Hospital, No. 1, West Section of Jinshajiang Avenue, Yibin, Sichuan, China; ^3^ Department of Mechanical and Electrical Engineering, Shenzhen Polytechnic University, Shenzhen, China; ^4^ Department of Rehabilitation Medicine, China Key Laboratory of Birth Defects and Related Diseases of Women and Children, Ministry of Education, West China Second University Hospital, Sichuan University, Chengdu, Sichuan, China; ^5^ Shenzhen Institute of Advanced Technology, Chinese Academy of Sciences, Shenzhen, China

**Keywords:** children with cerebral palsy, exoskeleton, gait recognition, IMU, deep learning

## Abstract

Accurate gait-phase identification in children with Cerebral Palsy (CP) constitutes a pivotal prerequisite for evidence-based rehabilitation. Addressing the precise detection of gait disturbances under natural ambulation, we propose a deep-learning framework that integrates a stacked denoising autoencoder (SDA) with a long short-term memory network (SDA–LSTM) to classify four canonical gait phases. A community-oriented dataset was constructed by synchronizing ankle-mounted inertial measurement units (IMU) with plantar-pressure insoles; natural gait sequences of six children with mild CP were acquired in open environments. The SDA layer robustly extracts discriminative representations from non-stationary, high-noise signals, whereas the LSTM module models inter-phase temporal dependencies, thereby enhancing generalization cross-user. In noise-free conditions the SDA–LSTM framework attained 97.83% accuracy, significantly exceeding SVM (94.68%), random forest (96.05%), and standalone LSTM (95.86%). Under additive Gaussian noise with SNR ranging from 5 to 30 dB, the model preserved stable performance; at 10 dB SNR (Signal-to-Noise Ratio), accuracy remained 90.96%, corroborating its exceptional robustness. These findings demonstrate that SDA–LSTM effectively handles the complex, heterogeneous gait patterns of children with CP and is readily deployable for clinical assessment and exoskeletal assistance systems, indicating substantial translational potential.

## 1 Introduction

CP represents the most prevalent motor disability in childhood (Wang N. et al., 2020). Primary dysfunctions manifest as movement disorders during postural control and locomotion, resulting in activity limitations (e.g., ambulation) (Hutton et al., 2000) (Armand et al., 2016). Children with CP exhibit characteristic gait deviations including prolonged stance phase, shortened swing phase, and reduced joint angle excursion amplitudes, significantly compromising mobility and quality of life. Clinically, precise quantification of gait phases and their dynamic progression constitutes a prerequisite for developing personalized rehabilitation protocols and evaluating interventional efficacy (Chang et al., 2010).

Motion capture systems have been extensively employed for whole-body kinematics and gait event detection in CP populations (Chang et al., 2010; Gage, 1993; Wishaupt et al., 2024; Damiano and Abel, 1996; Sutherland and Davids, 1993). However, conventional laboratory-based optical motion analysis faces limitations of high cost, spatial constraints, and inability to achieve long-term continuous monitoring in daily environments. Recent advances in wearable IMU offer new pathways for community-based gait analysis due to their miniaturization, cost-effectiveness, and integration capabilities (Zhang et al., 2018; Chen et al., 2016). Research confirms that ankle-worn IMUs effectively capture acceleration, angular velocity, and joint angle variations during gait cycles (Hutabarat et al., 2021), while coupling with plantar pressure signals further enhances gait event detection accuracy. Seel et al. developed an IMU-based joint angle measurement methodology for gait analysis (Seel et al., 2014). Nevertheless, high-amplitude non-stationary noise from motor control deficits in CP children, combined with inter-subject movement strategy heterogeneity, compromises feature extraction and generalization performance in traditional machine learning models. Achieving concurrent high robustness and cross-user consistency remains a critical scientific challenge in IMU-driven CP gait analysis.

The convergence of wearable sensors and deep learning provides innovative solutions. Behboodi et al. detected seven CP gait phases (Loading Response [LR], Mid-Stance [MSt], Terminal Stance [TSt], Pre-Swing [PSw], Initial Swing [ISw], Mid-Swing [MSw], Terminal Swing [TSw]) in real-time using dual gyroscopes (Behboodi et al., 2019). Lauer et al. achieved 95.3%–98.6% accuracy in gait event prediction via adaptive neuro-fuzzy inference systems (ANFIS) and supervisory control systems using lower-limb electromyography (EMG) (Lauer et al., 2005). Taborri et al. implemented hidden Markov models (HMM) with dual IMUs for biphasic gait recognition in CP subjects (Taborri et al., 2015). Yang et al. attained 95.53% accuracy in pediatric CP gait analysis through multimodal MRI-IMU-pressure data fusion with CNN-LSTM architectures (Yang et al., 2024; Wang L. et al., 2020). In prior work, we proposed a fusion framework integrating stacked denoising autoencoders with meta-learning for gait phase recognition, achieving 94.56% accuracy (Cao et al., 2024).

**FIGURE 1:**
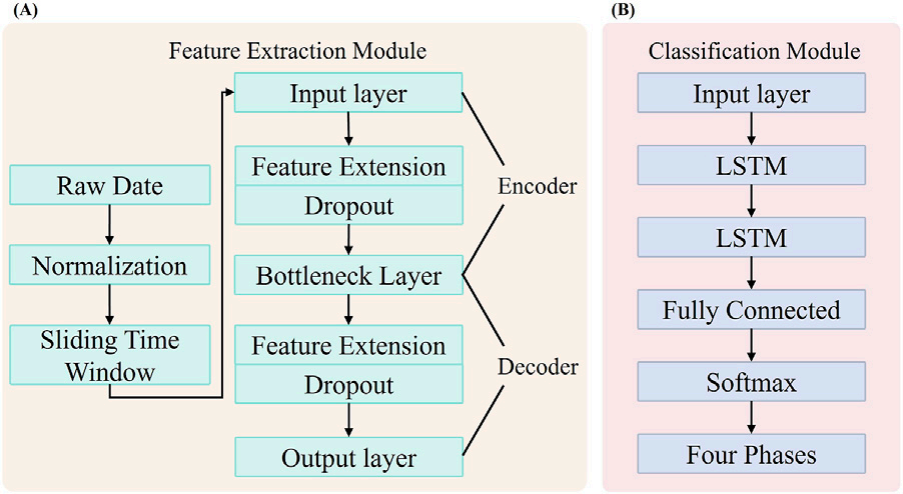
Framework for Gait Phase Recognition in Children with CP. **(A)** Feature extraction module based on a SDA. **(B)** Gait phase classification module for children with CP, implemented using a LSTM.

SDA enable unsupervised extraction of low-dimensional robust features while suppressing sensor drift and artifacts (Wang et al., 2024; Xiong et al., 2016) - we adapt them to model irregular CP gait patterns. LSTM networks excel at capturing long-range temporal dependencies (Luo et al., 2025; Yu et al., 2019) and have proven effective in healthy gait phase recognition. Building upon these foundations, this study proposes an SDA-LSTM fusion network for gait phase recognition in children with CP during unconstrained natural walking, with the analytical workflow illustrated in Figure 1.

The main contributions of this work are:

1. Construction of a synchronized ankle IMU-plantar pressure dataset for children with cerebral palsy in community-based open environments using a flexible ankle exoskeleton;
2. Design of an SDA-LSTM fusion network model, wherein the Stacked Denoising Autoencoder (SDA), incorporating Dropout regularization, “actively learns” abnormal movement patterns, and the Long Short-Term Memory (LSTM) network further models phase transition dynamics;
3. Systematic evaluation of model generalizability and robustness employing a dual strategy of cross-subject validation and multi-tiered noise injection.

## 2 Materials and methods

**FIGURE 2:**
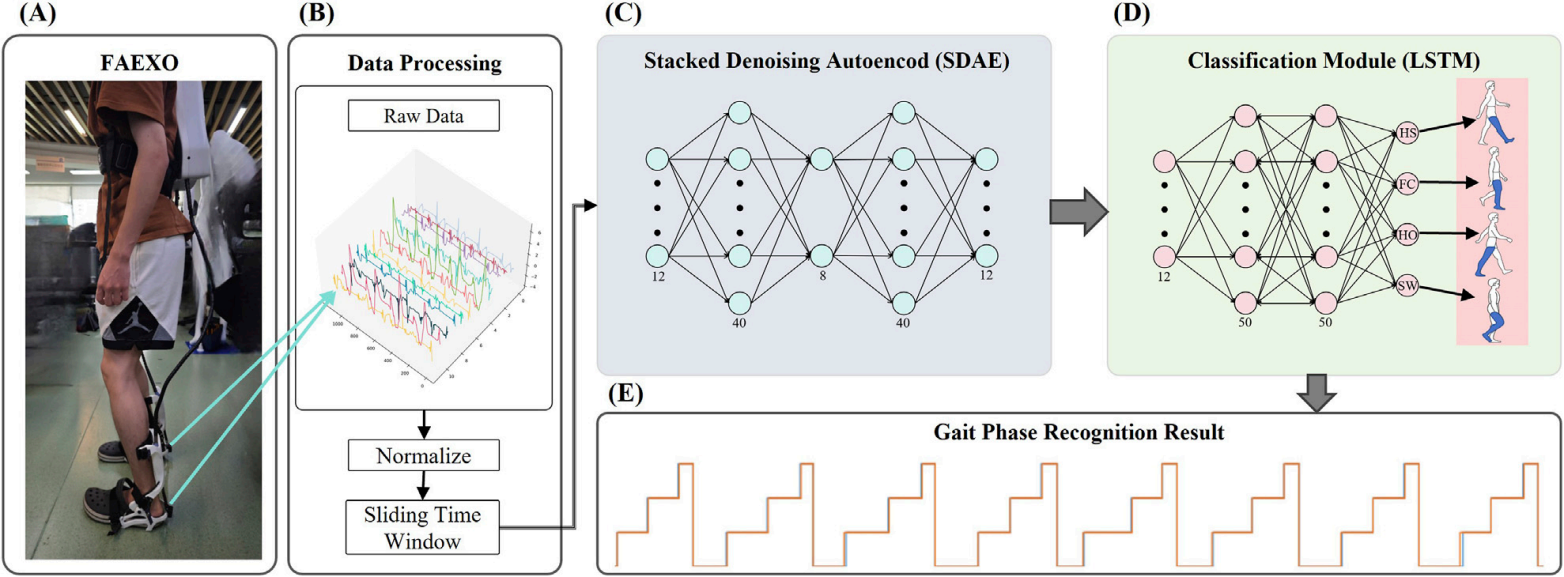
Architecture of the SDA-LSTM hybrid network. **(A)** Structural diagram of the flexible ankle exoskeleton (FAEXO). **(B)** Data preprocessing pipeline. **(C)** Schematic of the stacked denoising autoencoder (SDA), where the 12-dimensional output of the encoder serves as the input to the classification module. **(D)** LSTM architecture of the gait phase recognition module. **(E)** Gait phase (HS, FC, HO, SW) identification results generated by the SDA-LSTM hybrid network.

In this chapter, we will introduce the materials, methods, and specific implementation process used in the SDA-LSTM fusion network model for gait phase recognition in children with cerebral palsy. The Architecture of the SDA-LSTM hybrid network as shown in Figure 2.

### 2.1 Exoskeleton platform

The flexible ankle exoskeleton (FAEXO) employed in this study was designed by Ulon Robotics, with its specific structural configuration illustrated in Figure 2A. FAEXO represents an innovative rehabilitation assistive device specifically developed for children with CP. Its core design philosophy involves the decoupling of heavy components from actuation mechanisms, thereby achieving an optimal balance between lightweight wearability and precise torque assistance. The system comprises three principal components: a power backpack, a flexible transmission system, and an ankle joint module.

**FIGURE 3:**
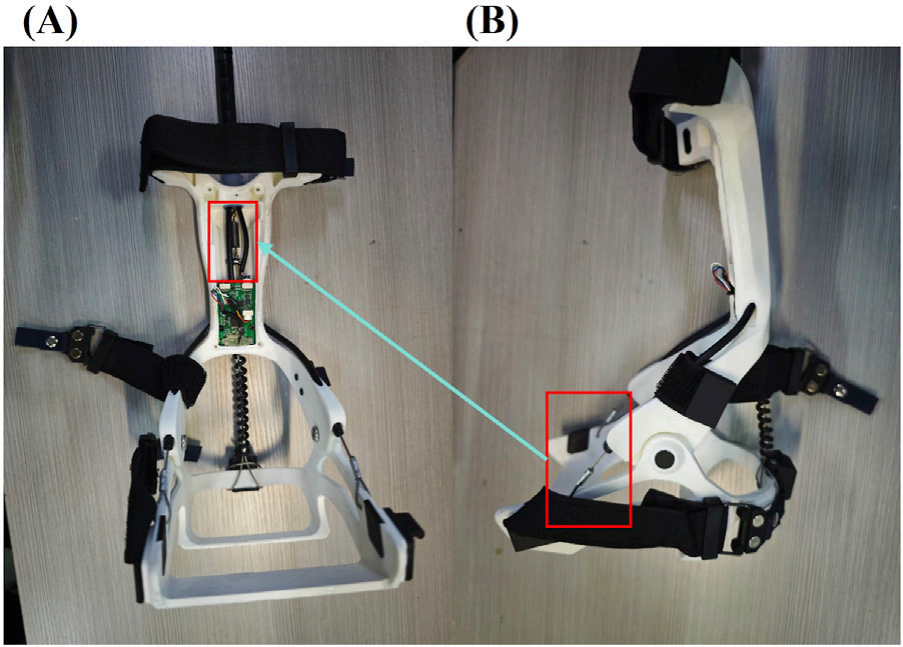
Schematic of the FAEXO ankle module; in panel. **(A)** Applying tensile assistance during the SW phase. **(B)** The red-framed steel cables connect to the fixed spring highlighted in the red frame of panel.

The powered backpack serves as the central control unit, integrating a miniature brushless motor, a high-precision MCU controller, and a detachable lithium battery. This configuration significantly reduces inertial loading on the lower limbs, avoiding interference with the child’s natural gait. The flexible transmission system employs pre-tensioned aerospace-grade stainless steel cables (2.0 mm diameter),sheathed within a spring and anchored to a TPU brace at the distal end of the lower leg, delivering assistive torque during heel-off. An embedded microcontroller, mounted superior to the calcaneus, processes IMU data from the heel and transmits it to the MCU. Posterior to the microcontroller, a stabilizing spring connects to the medial and lateral midfoot via two steel wires, applying an upward lifting force during the swing phase. The ankle joint structure is illustrated in Figure 3.

### 2.2 Data acquisition and pre-processing

Six ambulatory children diagnosed with mild cerebral palsy (CP) were recruited. Participants were partitioned into two cohorts: cohort A (n = 5) served as the training set, and cohort B (n = 1) as the validation set. All participants had previously done exoskeleton during over-ground walking to achieve habituation prior to data collection. The protocol was approved by the Institutional Review Board of the Pingshan County People’s Hospital (No. 20244142) and informed assents were obtained from all participants.

Data was acquired in an open, community-level environment. Throughout the experiment, participants walked on level ground at a self-selected, comfortable speed with minimal external constraints. Ankle kinematics were captured via two six-axis IMU embedded in the bilateral exoskeleton units, yielding tri-axial acceleration and angular velocity data. Sensors were positioned posterior to the calcaneus and sampled at 100 Hz. Plantar pressure signals were recorded using a dual-channel thin-film pressure sensor placed beneath the heel and first metatarsal head of the right foot, sampled at 50 Hz. The pressure signals were transmitted to a microcontroller via universal asynchronous receiver-transmitter (UART).

IMU signals acquired by the FAEXO system were processed in Python. Raw IMU data were filtered using a sixth-order Butterworth low-pass filter with a cut-off frequency of 100 Hz. Plantar pressure signals were binarized to identify four critical gait events on the right limb: heel strike, toe strike, heel off, and toe off. Gait phases were subsequently segmented according to these events.

### 2.3 Gait-phase segmentation

In most prior investigations, the gait cycle has been partitioned into four discrete phases: initial heel contact (H), flat-foot contact (F), push-off (or heel-off) (P), and subsequent limb swing (S) (Agostini et al., 2013; Rueterbories et al., 2010). Given that the present cohort comprises ambulatory children with mild cerebral palsy, this four-phase schema was retained.

Within this study, the gait cycle was segmented into four sequential locomotor stages according to the four critical gait events identified from plantar pressure signals. Specifically, the interval from initial heel strike to first toe contact was designated as the first phase, termed heel strike (HS). The second phase, spanning from toe contact to heel off, was defined as full contact (FC). The third phase, extending from heel off to toe off, was designated heels off (HO). The final phase, from toe off to the subsequent heel strike, constituted the swing phase (SP). A complete gait cycle was delimited from the first heel strike of phase 1 to the second heel strike of phase 4. In accordance with these definitions, each participant’s locomotor data was parsed into four contiguous gait phases, yielding a sequential trapezoidal profile of the gait cycle, as illustrated in Figure 2E.

## 3 Models and evaluation

This study proposes a hybrid neural architecture, SDA-LSTM, illustrated in Figure 2, for the recognition of gait phases in ambulatory children with paresis. Comparative experiments were conducted under both noisy and noise-free conditions against three benchmark models: support-vector machine (SVM), random forest (RF), and standard LSTM. SVM constructs an optimal hyperplane by maximizing the inter-class margin, yielding strong generalization in low-sample regimes (Jakkula, 2006; Chandra and Bedi, 2021). RF mitigates overfitting through the ensemble aggregation of multiple decision trees, demonstrating robustness against nonlinear, high-dimensional representations (Biau and Scornet, 2016; Breiman, 2001). The evaluation adheres to a rigorous, systematic protocol to provide a comprehensive appraisal of SDA-LSTM performance in the gait-phase classification task for children with cerebral palsy.

### 3.1 Network architecture

The proposed SDA-LSTM hybrid architecture integrates an SDA with LSTM network, comprising a feature-extraction module and a gait-phase classification module. The SDA extracts temporally resolved IMU features while enhancing model robustness; the LSTM captures spatially dependent local gait-phase patterns. This synergy enables simultaneous representation learning and gait-phase classification. The model architecture, illustrated in the figure, encompasses the following key components.

#### 3.1.1 Feature-extraction module

To effectively capture the dynamic characteristics embedded in the continuous, time-series gait signals throughout the gait cycle, the present study first applies z-score normalization to the 12-dimensional IMU inputs. Subsequently, a sliding-window strategy is employed to model temporal locality: a window length of 50 samples and a stride of 1 sample are selected, generating subsequences as specified in Equation 1.

$$X_{i}=\left\{x_{i},x_{i+1},\ldots ,x_{i+L_{w}-1}\right\}\in R^{L_{w}\times D}$$ (1)

Denotes the window length, and every frame possesses a feature dimensionality *D* (*D* = 12); the index i marks the starting position of the sliding window ( $1\leq i\leq T-L_{w}+1$ ). The stride s is set to unity to maximally preserve temporal continuity, and each window is assigned the classification label corresponding to its terminal sample. By this mechanism, the sliding procedure emulates the continuous evolution of gait, thereby augmenting the model’s temporal awareness and its capacity to extract dynamic features; the overlapping windows further effect a substantial expansion of the dataset. The data-processing pipeline is depicted in Figure 2B.

The pre-processed data are subsequently fed into the SDA network depicted in Figure 2C. The proposed SDA module is expressly designed to extract discriminative representations from the input signal; it is constructed by hierarchically stacking multiple denoising autoencoders (DAEs). Each DAE learns a noise-robust latent representation, enabling effective denoising and salient-feature extraction. The module comprises five principal layers: an input layer (12-D), a first expansion layer (40-D), a bottleneck layer (8-D), a second expansion layer (40-D), and an output layer (12-D). The encoder pathway, extending from the input layer to the bottleneck layer, compresses the raw sensor data into an eight-dimensional latent representation, whereas the decoder pathway, spanning from the bottleneck to the output layer, reconstructs the original input from this compressed code. To enhance robustness, dropout layers are interposed within both the encoder and decoder, injecting stochastic noise during training (Srivastava et al., 2014).

In the proposed SDA-LSTM hybrid model, the SDA functions as a dedicated feature extractor, comprising an encoder–decoder architecture. The encoder projects the input into a low-dimensional latent space via two fully connected layers, corresponding to the input layer and the first expansion layer. During training, dropout (rate = 0.2) is incorporated to enhance robustness. Formally, for an input vector x, the encoding operation is defined as Equation 2:

$$h=f_{\theta }\left(x\right)=\sigma \left(W_{1}x+b_{1}\right)$$ (2)

Where *θ* denotes the parameter set of the encoder, and *σ* denotes the activation function (ReLU).

The decoder reconstructs the original input from the low-dimensional representation; it is likewise implemented as two fully connected layers that correspond to the second expansion layer and the output layer. The decoding transformation is expressed as Equation 3:

$$\hat{x}=g_{\theta^{\prime }}\left(h\right)=\sigma \left(W_{2}h+b_{2}\right)$$ (3)

*θ′* denotes the parameter set of the decoder.

#### 3.1.2 Gait-phase recognition module

The gait-phase recognition module is implemented with an LSTM network, depicted in Figure 2D. Long Short-Term Memory constitutes a specialized recurrent architecture capable of capturing long-range temporal dependencies, thereby alleviating the gradient vanishing or explosion issues endemic to conventional RNNs. Training proceeds through forward and backward propagation: during the forward pass, each LSTM cell updates its cell state and computes its output contingent on the current input and the preceding hidden state; during the backward pass, gradients are computed, and the parameters are updated via an optimizer. In this study, the LSTM comprises an input layer, two hidden layers each containing 50 units, and a fully connected output layer. The network receives the 12-dimensional feature sequences extracted by the SDA and predicts the label of the first time-step immediately following each sliding window. A final fully connected layer maps the latent representation to a four-dimensional class space corresponding to the four gait phases (HS, FC, HO, SW), thereby accomplishing the gait-phase recognition task for the pediatric participants.

### 3.2 Model training

**FIGURE 4:**
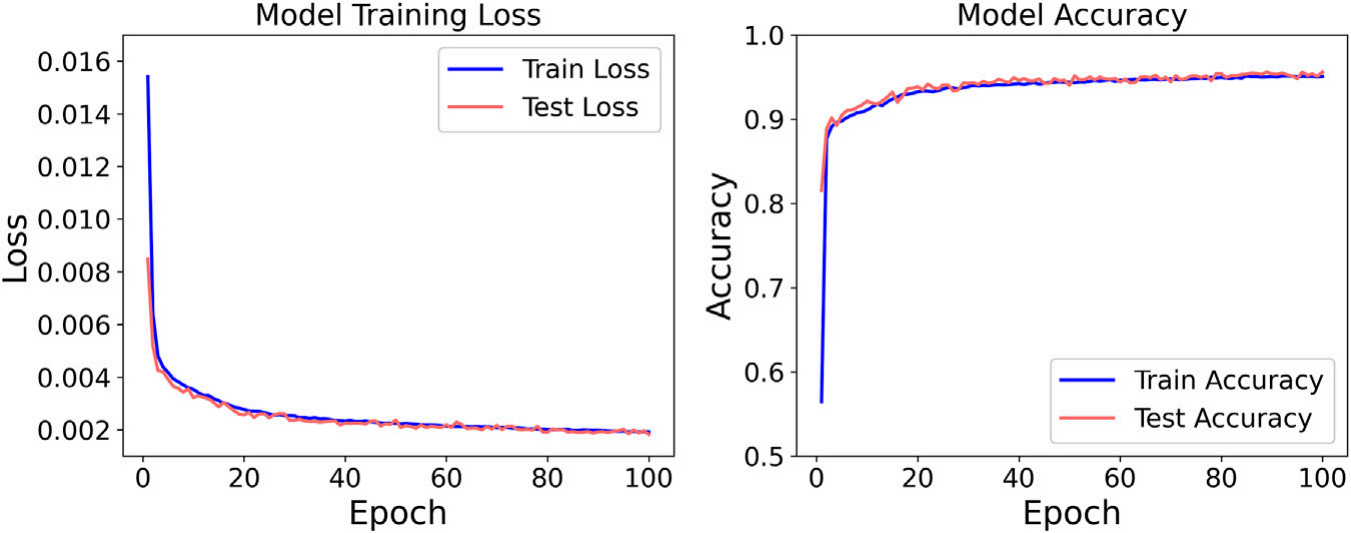
Loss and accuracy of SDA-LSTM in classification tasks.

Consistent with the previously outlined protocol, the dataset was bifurcated into cohort A (n = 5) and cohort B (n = 1). Model training was exclusively conducted on cohort A, employing five-fold cross-validation. Specifically, the data were randomly divided into five mutually exclusive subsets; in each fold, four subsets (80%) were used for training and the remaining subset (20%) for validation, iterating until every subset had served as the validation set once. Hyper-parameters were held constant across folds, and the average training loss and validation loss of the five resulting models were computed to assess hyper-parameter quality. Once optimal hyper-parameters were identified, the entire cohort A was leveraged as the training set. After model convergence, the unseen data from the single participant in cohort B were used for external validation; predictions generated by the model were compared against ground-truth labels to evaluate generalizability. The entire network was implemented in Python 3.11.7 using PyTorch 2.3.1. Each model was trained for 100 epochs with an initial learning rate of 0.0001. The corresponding training loss and accuracy trajectories are presented in Figure 4.

The comprehensive training protocol of the network model encompasses two sequential phases. Phase one is executed within the feature extraction module. The proposed gait-feature extraction module adopts a cascaded processing architecture. Initially, raw six-dimensional IMU data undergo global normalization via Min-Max Scaling, whereby each feature dimension is linearly mapped onto the interval [0, 1] to eliminate dimensional disparities. Subsequently, a sliding window of fixed length 50 and stride 1 segments the normalized time-series data, and an overlapping sampling strategy is employed to construct spatio-temporal feature matrices. These matrices are then fed into a stacked denoising autoencoder (SDA) for deep feature learning, yielding 12-dimensional reconstructed features. Throughout the encoding and decoding stages, Dropout layers (p = 0.2) and ReLU activation functions are incorporated to emulate noise. Within this framework, the loss function of the SDA, given N samples, is defined by the ReLU formulation presented in Equation 4.

$$L_{\text{SDA}}=\frac{1}{N}\sum_{i=1}^{N}{\left\|x_{i}-{\hat{x}}_{i}\right\|}^{2}$$ (4)

*N* denotes the batch size.

The second stage encompasses the classification module, namely, the LSTM-based phase-classification network. This module leverages a two-layer LSTM that ingests the 12-dimensional features generated by the SDA and executes gait-phase classification. Within this study, the over-ground gait cycle is delineated into four discrete phases:HS, FC, HO, and SW,encoded as labels 0, 1, 2, and 3, respectively, and these labels constitute the target outputs of the network. Temporal modeling via the LSTM is formally expressed as Equation 5:

$$h_{T}=\text{LSTM}\left(h_{1},...,h_{50}\right)$$ (5)

During training, the network is optimized by minimizing the categorical cross-entropy loss, specified in Equation 6.

$$L=-\sum_{k=1}^{4}y_{k}\log P\left(y=k|h_{T}\right)$$ (6)

Using the Adam optimizer. Adam dynamically adjusts parameter updates through adaptive moment estimation (Pang et al., 2026), thereby balancing gradient direction and magnitude; its update rule is given by Equation 7:

$$\theta_{t+1}=\theta -\eta \cdot \frac{{\hat{m}}_{t}}{\sqrt{{\hat{v}}_{t}}+\epsilon }$$ (7)

Let η denote the learning rate (lr = 5 × 10^−5^ in the code), ε a small constant to prevent division-by-zero (commonly 1 × 10^−8^), is the first-moment estimate (mean), and is the second-moment estimate (uncentered variance).

The final classification layer employs a softmax function to yield a categorical probability distribution. A fully-connected layer projects the latent representation into a four-dimensional class space corresponding to the gait phases HS, FC, HO, and SW. The softmax expression is specified in Equation 8.

$$P\left(y=k|h_{T}\right)=\frac{\exp\left(w_{k}^{T}h_{T}+b_{k}\right)}{\sum_{j=1}^{4}\exp\left(w_{j}^{T}h_{T}+b_{j}\right)}$$ (8)

Where $h_{T}\in R^{d}$ denotes the hidden-state vector of the LSTM network at the final time step, $w_{k}\in R^{d}$ is the weight vector corresponding to the k-th class, and $b_{k}\in R$ is the class-specific bias term.

### 3.3 Benchmark models

To rigorously validate the efficacy of the proposed SDA-LSTM fusion network for gait-phase recognition in children with CP, three classical machine-learning algorithms were adopted as comparative baselines: SVM, random forest (RF), and a standalone LSTM network. Collectively, these baselines epitomize traditional machine learning, ensemble learning, and deep learning paradigms, respectively, and are widely recognized for their strong empirical performance across diverse domains.

### 3.4 Model evaluation

Classification accuracy constitutes the primary metric for evaluating gait-phase recognition capacity. Model performance is therefore quantified via a two-tier accuracy framework encompassing (i) overall gait-phase recognition accuracy and (ii) class-specific accuracies for each individual phase. Performance across the four gait phases is visualized via confusion matrices. Overall Accuracy (OA) is formally expressed as Equation 9:

$$Accuracy=\frac{\sum_{i=1}^{K}N_{ii}}{N}$$ (9)

*K* denotes the number of gait-phase classes, *Nii* represents the count of correctly classified samples for class, and *N* indicates the total sample size.

## 4 Results

To rigorously validate the efficacy of the proposed methodology, the four models were evaluated under cross-user and six-level-noise conditions via two complementary experiments: gait-phase recognition and robustness testing. All experimental protocols were executed in strict adherence to scientific standards to ensure reliability and validity.

### 4.1 Accuracy of motion-intention recognition under cross-user conditions

**FIGURE 5:**
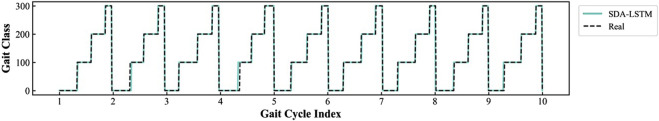
Comparison between the SDA-LSTM model outputs and the ground-truth gait labels under cross-user conditions.

Based on the ankle joint IMU and gait phase database we established for CP patients, we conducted cross-user experiments to validate the SDA-LSTM fusion model proposed in this study. Figure 5 shows a visual comparison of the model’s predicted results and the actual results.

**FIGURE 6:**
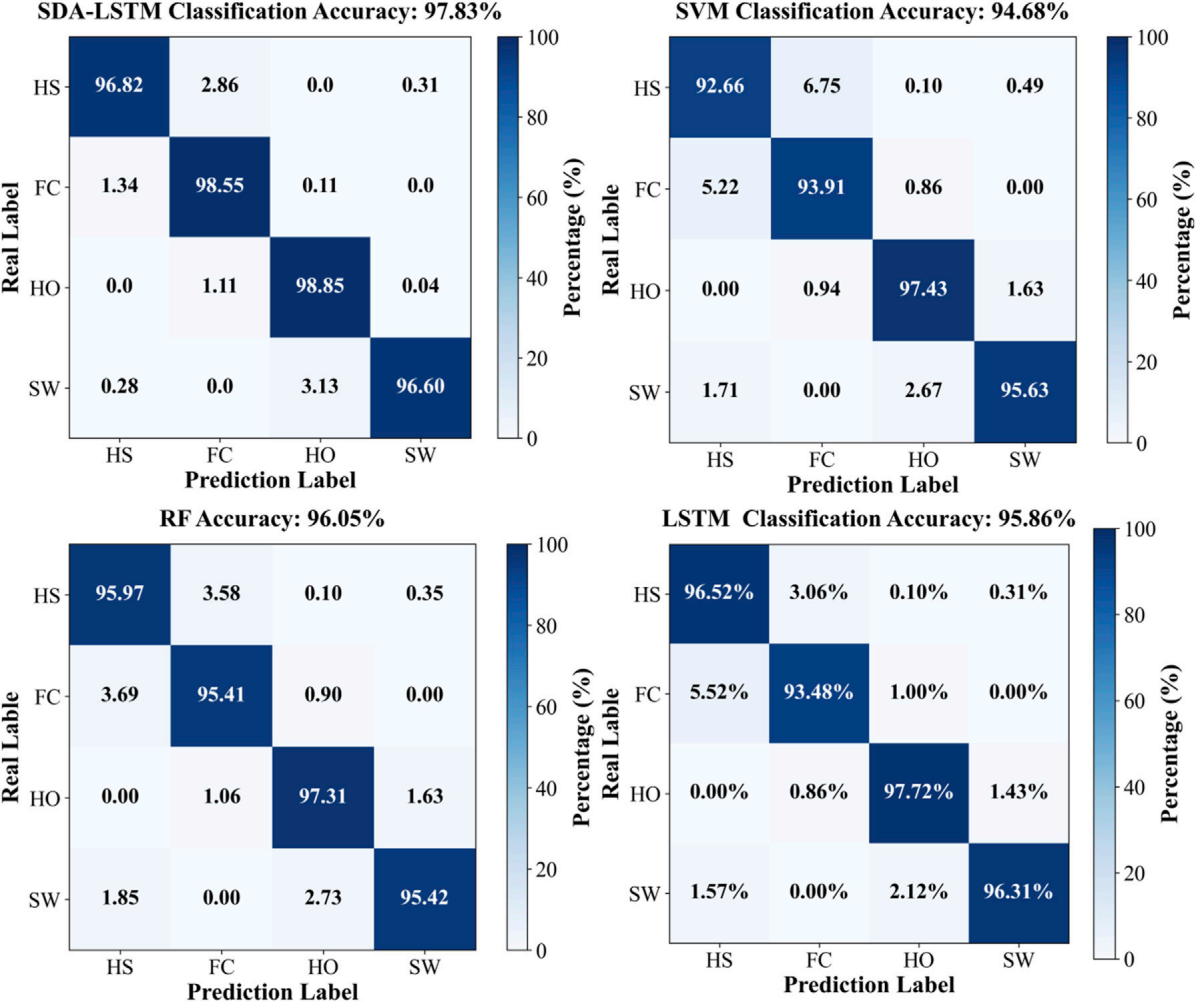
Confusion matrices for the four models (SDA-LSTM, SVM, RF, LSTM), illustrating classification performance across the four gait phases (HS, FC, HO, SW).

Under cross-user conditions, the proposed SDA-LSTM fusion model was benchmarked against SVM, RF, and LSTM. Across the four gait-phase categories, SDA-LSTM attained higher class-wise and overall accuracies than the comparative models. The overall accuracies for SDA-LSTM, SVM, RF, and LSTM were 97.83%, 94.68%, 96.05%, and 95.86%, respectively. The recognition performance of the four models for each gait phase is depicted via confusion matrices in Figure 6.

**FIGURE 7:**
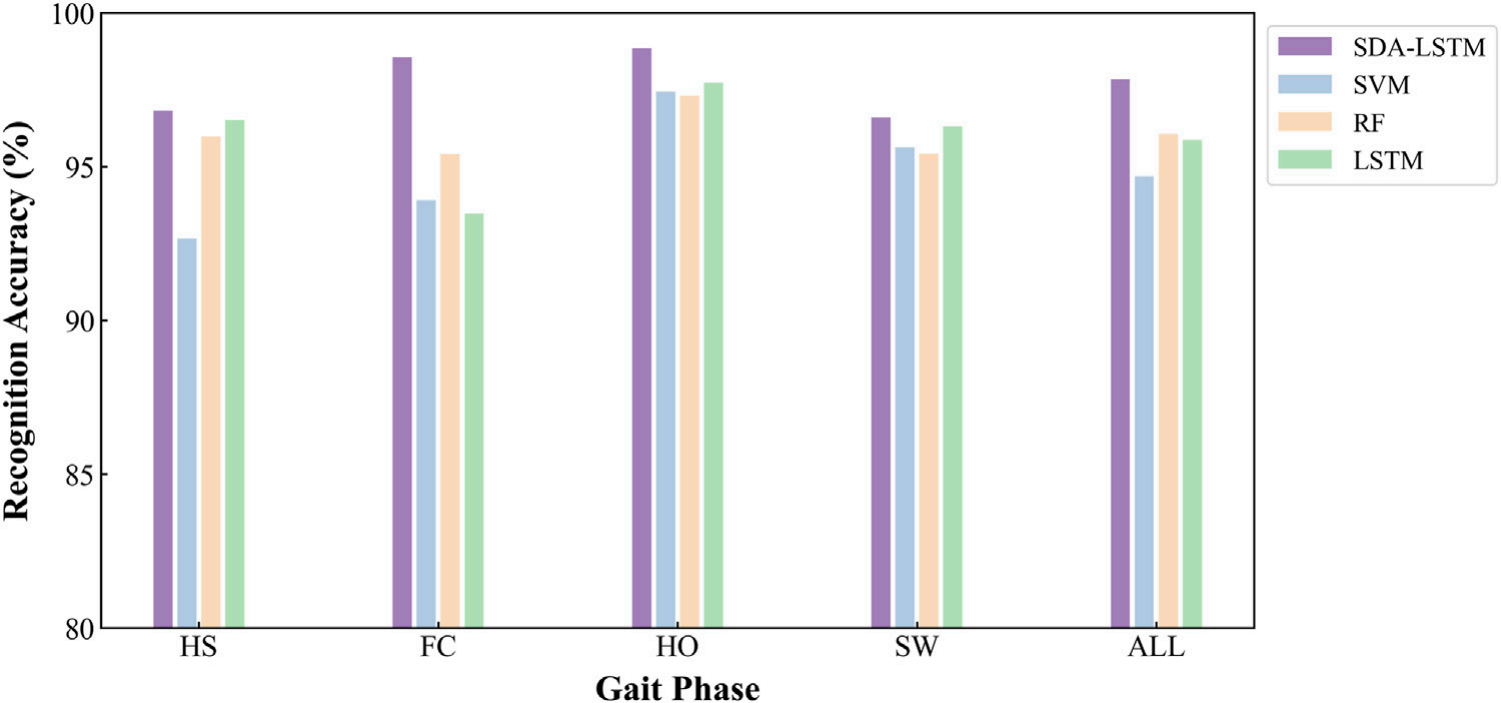
Employs bar charts to compare the cross-user recognition performance of the four algorithms:DA-LSTM, SVM, RF, and LSTM, on the four gait phases.

Specifically, the rows of the confusion matrix denote the ground-truth labels, and the columns denote the predicted labels; the four classes are HS, FC, HO, and SW. In the proposed SDA-LSTM fusion model, among samples whose true label is HS, 96.82% were correctly classified, only 2.86% were misclassified as FC, 0.31% were misclassified as SW, and none were misclassified as HO. Among samples whose true label is FC, 98.55% were correctly classified, 1.34% were misclassified as HS, 0.11% were misclassified as HO, and none were misclassified as SW. Among HO samples, 98.85% were correctly classified, 1.11% were misclassified as FC, 0.04% were misclassified as SW, and none were misclassified as HS. In the SW class, 96.60% were correctly classified, 3.13% were misclassified as HO, 0.28% were misclassified as HO, and none were misclassified as FC. The per-phase recognition accuracies of SDA-LSTM, SVM, RF, and LSTM under cross-user conditions are reported in Table 1. We have plotted bar charts to demonstrate the recognition accuracy of the four models across the four gait phases, as shown in Figure 7.

**TABLE 1:** Accuracy rates of four gait phase recognition models (SDA-LSTM, SVM, RF, and LSTM) under across user conditions.

Model	HS	FC	HO	SW
SDA-LSTM	96.82%	98.55%	98.85%	96.60%
SVM	92.66%	93.91%	97.43%	95.63%
RF	95.97%	95.41%	97.31%	95.42%
LSTM	96.52%	93.48%	97.72%	96.31%

### 4.2 Model robustness analysis

This experiment quantitatively investigates the robustness of the SDA-LSTM model against noise perturbations during gait-phase classification. Contamination severity was systematically manipulated by injecting additive white Gaussian noise (AWGN) into the cross-subject test data under offline conditions; model performance was then evaluated at six noise levels and compared with the pristine (noise-free) baseline. AWGN, ubiquitous in signal processing and deep learning, serves as a canonical surrogate for sensor inaccuracies, channel interference, or environmental disturbances. Concretely, the SciPy-signal library’s awgn routine was employed to inject AWGN into each input sequence. The signal-to-noise ratio (SNR), a widely adopted metric quantifying the relative power of the useful signal to background noise, is defined in Equation 10:

$$\text{SNR }\left(\text{dB}\right)=10\log_{10}\left(\frac{P_{\text{signal}}}{P_{\text{noise}}}\right)$$ (10)

*P*_signal_ denotes signal power and *P*_noise_ denotes noise power. AWGN was injected at six discrete SNR levels:5, 10, 15, 20, 25, and 30 dB, corresponding to noise-to-signal power ratios of 1:3.16, 1:10, 1:31.6, 1:100, 1:316, and 1:1,000, thereby spanning the continuum from “severely perturbed” to “nearly pristine.” Labels remained unchanged across all levels, and the overall accuracy of SDA-LSTM was computed for each SNR condition. Signal-to-noise ratio (SNR), defined as the power ratio between the clean signal and the injected noise, serves as the pivotal robustness metric; a lower SNR denotes stronger noise. The entire protocol was executed in a Python environment.

**FIGURE 8:**
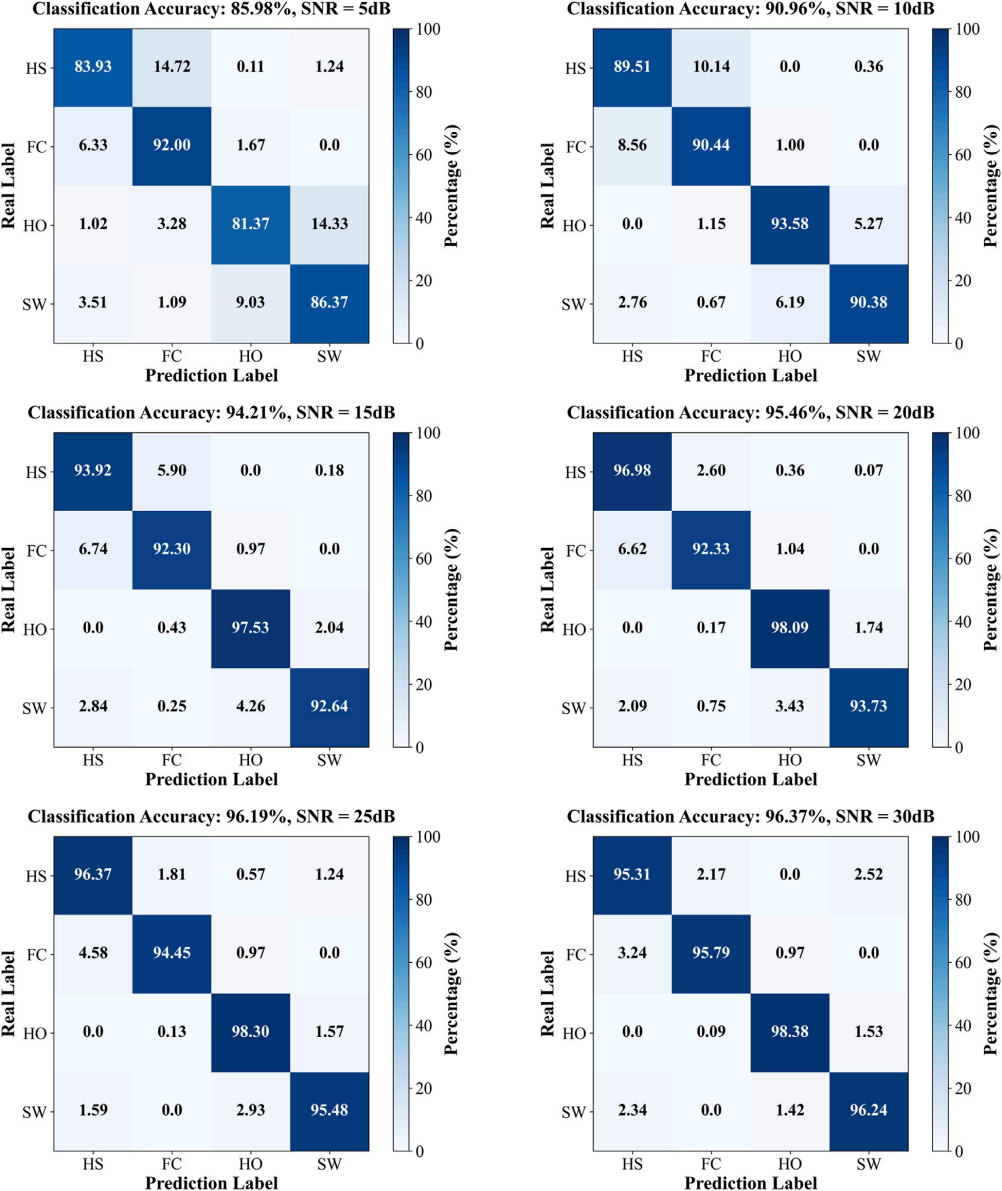
Confusion matrices of SDA-LSTM under six distinct SNR conditions (5, 10, 15, 20, 25, 30 dB).

AWGN was applied to the data of the single participant in cohort B, and the corrupted sequences were subsequently evaluated by the SDA-LSTM fusion network. Recognition accuracies at SNR = 5, 10, 15, 20, 25, and 30 dB were 85.98%, 90.96%, 94.21%, 95.46%, 96.19%, and 96.37%, respectively. The corresponding confusion matrices for SDA-LSTM are presented in Figure 8.

The per-phase recognition accuracies of the SDA-LSTM model under the six noise conditions and the noise-free baseline are reported in Table 2.

**TABLE 2:** Accuracy of gait phase recognition in six noisy and no noise cases of gait phase.

SNR (dB)	HS	FC	HO	SW	All
5	83.93%	92%	81.37%	86.37%	85.98%
10	89.51%	90.44%	93.58%	90.38%	90.96%
15	93.92%	92.3%	97.53%	92.64%	94.21%
20	97.33%	92.33%	98.09%	93.37%	95.46%
25	98.15%	94.45%	98.3%	95.48%	96.19%
30	97.76%	95.79%	98.38%	96.24%	96.37%
NO Noise	96.82%	98.55%	98.85%	96.6%	97.83%

**FIGURE 9:**
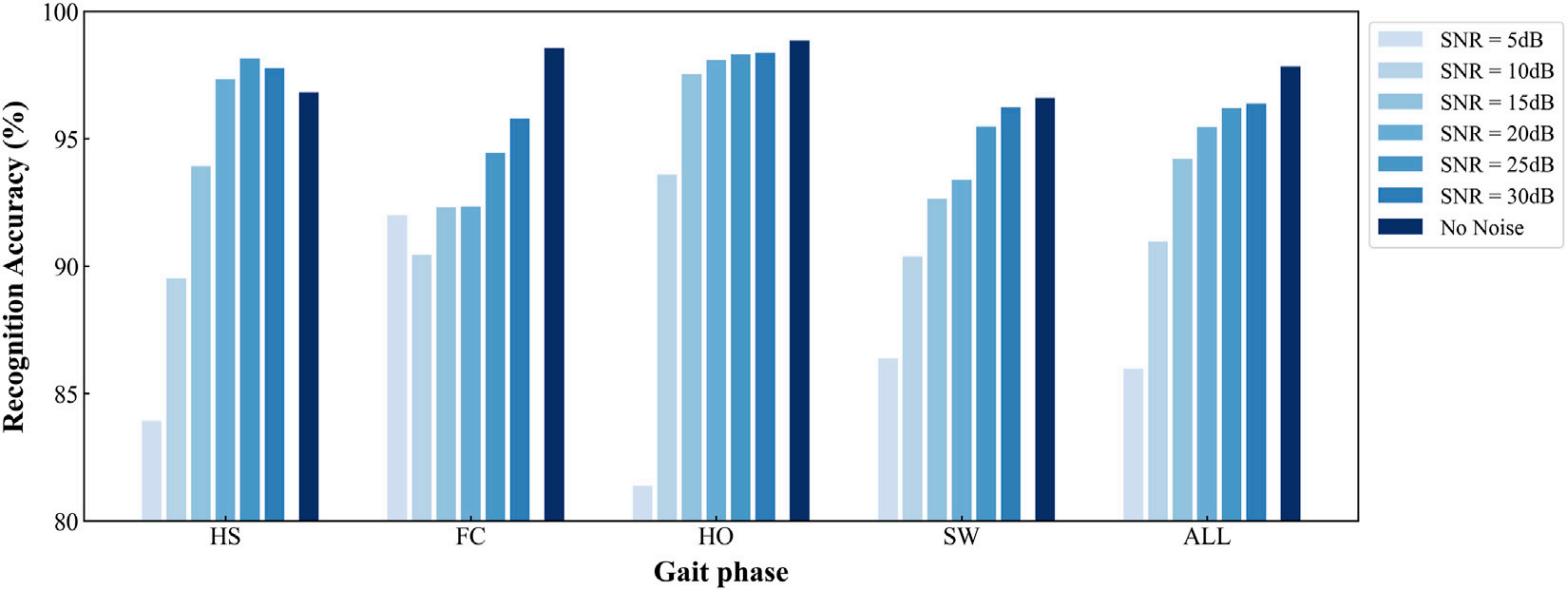
Per-phase and overall accuracies of SDA-LSTM across six noise levels and a noise-free baseline.

To visualize the accuracy trajectories of the four algorithms across identical SNR levels within a single model, we constructed a bar plot (Figure 9). Within each algorithmic panel, bar intensities deepen monotonically with increasing data purity (i.e., decreasing noise), thereby enabling an unambiguous demonstration of the SDA-LSTM model’s superior performance in the present task.

The developed SDA-LSTM model achieved gait-phase recognition accuracies of 85.98%, 90.96%, 94.21%, 95.46%, 97.19%, 96.37%, and 97.83% under additive white Gaussian noise at SNR = 5, 10, 15, 20, 25, 30 dB, respectively, as well as under pristine conditions.

**FIGURE 10:**
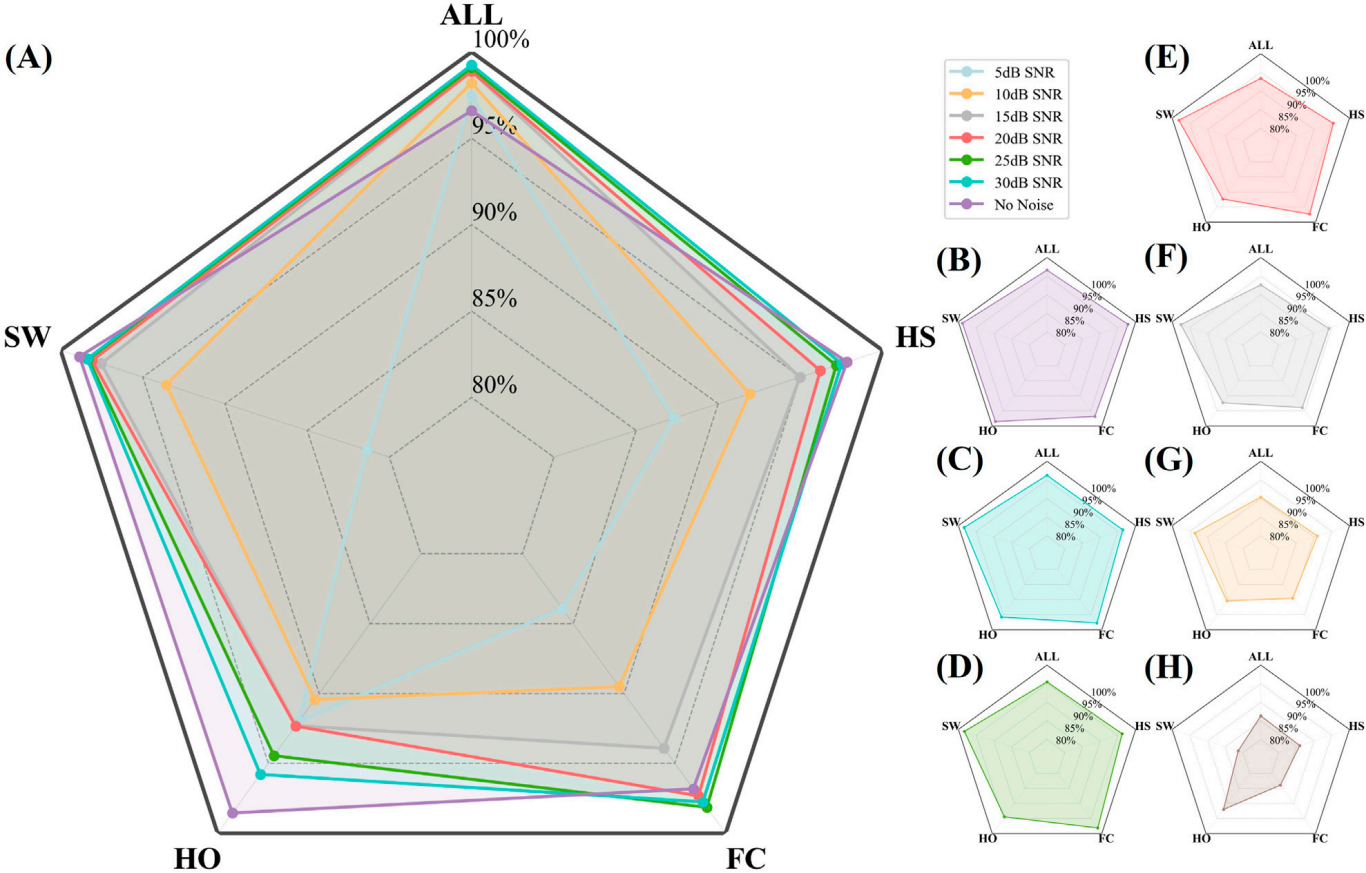
Radar plot illustrating the per-phase (HS, FC, HO, SW) and overall accuracies of SDA-LSTM under six SNR levels (5, 10, 15, 20, 25, 30 dB) and noise-free conditions. **(A)** Recall rates and overall accuracy for four gait phases under six noise conditions and no noise. **(B–H)** Represent recall rates and overall accuracy for four gait phases under no noise and SNR conditions of 30, 25, 20, 15, 10, and 5 dB, respectively.

To provide an intuitive visualization of the SDA-LSTM fusion model’s performance across the six noise levels, a radar chart systematically compares the per-phase accuracies for HS, FC, HO, SW and the overall accuracy at SNR = 5, 10, 15, 20, 25, 30 dB and under noise-free conditions (Figure 10). Joint analysis of the radar plot and Table reveals that accuracy exceeds 95% whenever SNR >15 dB and surpasses 90% whenever SNR >10 dB.

## 5 Discussion

### 5.1 Demonstrates robust cross-user generalization

This study rigorously benchmarked the SDA-LSTM architecture against three reference models: SVM, RF, and LSTM using an external dataset to quantify cross-user recognition accuracy in children with cerebral palsy. The proposed SDA-LSTM achieved 97.83% accuracy, surpassing SVM (94.68%) by 3.15 percentage points, RF (96.05%) by 1.78 percentage points, and standalone LSTM (95.86%) by 1.97 percentage points. These margins underscore the pronounced superiority of deep-learning-based approaches over conventional machine-learning paradigms.

Gait pathologies in CP present highly non-linear spatio-temporal dynamics; the SDA-LSTM successfully captured spastic-type prolongations of the stance phase as well as athetoid-type trajectory tremors within the swing phase, thereby mitigating phase-boundary misclassifications that afflict SVM and RF due to their inherent limitations in manual feature engineering. Although LSTM inherently accommodates temporal sequences, it inadequately models cross-phase coupling features induced by fluctuating muscle tone. By leveraging hierarchical memory units, SDA-LSTM strengthens long-range dependency learning, markedly enhancing robustness at transition points across the gait cycle.

Across all four phases: HS, FC, HO, and SW. SDA-LSTM delivered the highest recognition accuracies relative to SVM, RF, and LSTM, corroborating the efficacy of temporal networks for gait-phase identification and demonstrating that the proposed SDA-LSTM retains commendable accuracy and generalizability under cross-user deployment scenarios.

### 5.2 Maintains elevated accuracy across multi-level noise perturbations

Owing to the intrinsic pathological complexity of cerebral palsy, children with CP exhibit involuntary movements, abnormal co-contractions, and dynamic fluctuations in muscle tone during ambulation, all of which severely distort the kinematic trajectories of the lower limbs and manifest as high-amplitude noise in the sensor stream. To contend with these phenomena, the proposed SDA module incorporates a Dropout mechanism (p = 0.2) that actively emulates the abrupt, pathophysiology-driven signal disturbances encountered during walking. Validation was performed by injecting additive noise into the raw data of cohort B, thereby permitting a systematic evaluation of SNR-dependent effects on the recognition accuracy of each of the four gait phases and on the overall classification rate. Results demonstrate that the SDA-LSTM model maintains a phase-recognition accuracy of 90.96% at an SNR of 10 dB,a degradation of only 6.87% relative to the noise-free baseline,and retains 85.98% accuracy even under severe noise (SNR = 5 dB, ≈3.16 : 1 signal-to-noise ratio). Bar plots further reveal that noise-induced performance loss exhibits pronounced phase dependency and non-linear decay characteristics. Specifically, at an SNR of 5 dB, the HO phase, whose discrimination relies on subtle joint-angle cues such as the peak knee-flexion angle, experiences a precipitous accuracy drop to 81.37%, thereby constituting the primary vulnerability to noise contamination.

### 5.3 Future work

Nevertheless, although the present SDA-LSTM architecture has demonstrated commendable performance in cross-user and robustness evaluations, its translation to clinical utility confronts multifaceted challenges. In forthcoming work, we will prioritize multi-modal sensor fusion, integrating IMU and plantar-pressure signals,to augment discriminative capacity, and we will conduct real-time validation with an exoskeleton in ecologically valid settings, thereby furnishing both empirical evidence and theoretical foundations for clinical assessment and ultimately enabling precise quantification and active remediation of gait dysfunction in children with cerebral palsy.

## 6 Conclusion

Motivated by the clinical imperative for precise gait-assessment in children with cerebral palsy (CP), this study proposes a hybrid SDA-LSTM framework for gait-phase identification. Leveraging dual-modal signals acquired from IMUs and plantar-pressure sensors embedded within a soft ankle–foot exoskeleton, natural walking data were collected in an open community environment to classify four discrete gait phases (HS, FC, HO, SW). Six ambulatory children with CP were recruited, and a cross-subject validation protocol was adopted to examine generalizability. Relative to SVM, RF, and LSTM baselines, the SDA-LSTM model achieved an overall accuracy of 97.83% under noise-free conditions, surpassing SVM (94.68%), RF (96.05%), and LSTM (95.86%). Even under stringent noise (SNR = 10 dB), the model retained 90.96% accuracy,a degradation of only 6.87% relative to the clean condition,and maintained 85.98% accuracy at SNR = 5 dB (≈3.16 : 1 signal-to-noise ratio), underscoring its pronounced robustness. These findings demonstrate that the SDA-LSTM framework effectively mitigates the non-linear and non-stationary characteristics inherent in the aberrant locomotor patterns of children with CP, thereby furnishing a reliable algorithmic foundation for clinical gait quantification and proactive intervention.

## Data Availability

The original contributions presented in the study are included in the article/supplementary material, further inquiries can be directed to the corresponding authors.
